# The molecular basis of brain injury in preterm infants with sepsis - associated encephalopathy

**DOI:** 10.1186/s12887-022-03372-5

**Published:** 2022-06-10

**Authors:** Xiaohui Gong, Bowen Weng, Xiaoyue Zhang, Chongbing Yan, Cheng Cai

**Affiliations:** Department of Neonatology, Shanghai Children’s Hospital, School of medicine, Shanghai Jiao Tong University, No. 355, Luding Road, Shanghai, 200062 People’s Republic of China

**Keywords:** Sepsis-associated encephalopathy, Brain injury, Preterm infants, Molecular basis, microRNAs

## Abstract

Sepsis-associated encephalopathy (SAE) is characterized by brain dysfunction during sepsis, without central nervous system infection. Here, we explored the molecular basis of brain injury in preterm infants with SAE. From Jan 2016 to Dec 2019, a total of 20 preterm infants were hospitalized in the neonatal intensive care unit (NICU) of our hospital, including 10 preterm infants with SAE (SAE group) and 10 preterm infants without encephalopathy after sepsis (no SAE group). Among the 20 premature infants, there were 12 males and 8 females, with mean gestational age 31.0 ± 2.46 weeks, 7 cases with birth weight ≤ 1500 g and 13 cases with birth weight 1500–2500 g. Blood cultures were negative in 6 cases and positive in 14 cases, including 10 cases of Gram-negative and 4 cases of Gram-positive bacteria, respectively. Expression levels of messenger RNA (mRNA) and MicroRNA (miRNA) were analyzed in peripheral blood samples from both groups during sepsis. There were 1858 upregulated and 2226 downregulated mRNAs [fold-change (FC) > |2|, *p* < 0.05], and 322 upregulated and 160 downregulated miRNAs (FC > |2|, *p <* 0.05), respectively, in the SAE group compared with the no SAE group. Expression levels of miRNA-1197 [95% confidence intervals (CI), 0.042 to 0.166] were 6.03-fold higher in the SAE group than the no SAE group, while those of miRNA-485-5p (95% CI, 0.064 to 0.024) were lower (0.31-fold). Both high expression of miRNA-1197 and low expression of miRNA-485-5p may be associated with pathogenic alteration of the oxidative respiratory chain and energy metabolism in preterm infants with SAE.

**Supplementary Information:**

The online version contains supplementary material available at 10.1186/s12887-022-03372-5.

## Introduction

Sepsis is a dysregulated systemic response to infection that causes very high rates of morbidity and mortality worldwide [1]. Complications that can develop from sepsis include septic shock and multiple organ dysfunction syndrome (MODS), which are among the most important causes of morbidity and mortality in the neonatal intensive care unit (NICU) [2]. Sepsis-associated encephalopathy (SAE) is defined as a diffuse or multifocal cerebral dysfunction induced by the systemic response to the infection without clinical or laboratory evidence of direct brain infection. Its pathogenesis is multifactorial. SAE generally occurs early during severe sepsis and precedes multiple-organ failure [3]. The pathogenesis of SAE in premature infants remains unclear and may involve dysfunction of the blood–brain barrier (BBB), inflammatory factors, oxidative stress (OS), apoptosis, and mitochondrial dysfunction.

The neurological prognosis of preterm infants after sepsis is highly variable. Not all preterm infants develop SAE after sepsis, suggesting that differences in genetic signatures may influence the nervous system response to systemic inflammatory response syndrome (SIRS) in these babies. SIRS is a group of clinical symptoms caused by systemic non-specific inflammatory responses, triggered by serious injury of the body caused by various events, such as infection, trauma, burn, operation, and ischemia-reperfusion, which eventually leads to an uncontrolled inflammatory response.

Functional analysis of nervous system microRNAs (miRNAs) is a highly active new field of investigation [4]. The maturation, migration, and myelination of neurons in the nervous system of preterm infants are regulated by miRNAs [5]. In order to better understand the molecular basis of SAE in premature infants, this study analysed expression levels of mRNA and miRNA in peripheral blood samples from infants with SAE and infants without encephalopathy after sepsis.

## Materials and methods

### Subjects and sample collection

Premature infants with (*n* = 10) and without (*n* = 10) SAE after sepsis, according to standard diagnosis criteria for SAE and sepsis, were recruited in the NICU of Shanghai Children’s Hospital, from January 2016 to December 2019. Infants included in this study were premature and had neonatal respiratory distress syndrome (NRDS). Perinatal infection was detected in 15 of the 20 infants, with admission time within 12 h after birth.

The Shanghai Children’s Hospital Ethics Committee approved our research. After obtaining signed informed consent from guardians of the patients, we collected venous blood and cerebrospinal fluid (CSF) samples from the premature infants. Venous blood was collected from the femoral vein of all 20 premature infants at 12 h after admission and CSF was collected approximately 24 h after diagnosis of sepsis.

### Premature infant SAE inclusion and exclusion criteria

The inclusion criteria for premature infants with SAE were premature infants of gestational age < 37 weeks. The definitive diagnostic criteria for sepsis are clinical manifestations (For example, there may be reduced milk intake or milk refusal, milk overflow, drowsiness or irritability, low crying, fever or body temperature, or pale face, depressed expression, low response, etc.,) and any of the following: 1) pathogenic bacteria cultured from blood and aseptic body samples; 2) opportunistic pathogens identified in blood culture and the same bacteria were cultured from either a second blood sample, a sterile body cavity sample, or a sample from a catheter head. Clinical diagnosis of sepsis was based on clinical manifestations and any of the following: 1) two or more non-specific tests for inflammatory markers [For example, white blood cell (WBC), proportion of neutrophils, acute C-reactive protein (CRP), procalcitonin (PCT), Interleukin-6 (IL-6), etc.], and 2) positive blood test for pathogenic bacterial antigen or DNA. If the results of lumbar puncture examination of CSF are as follows, WBC of CSF is < 20 × 10^6^/L, protein < 1000 mg/L, simultaneous peripheral blood trace glucose > 50%, negative blood culture, so that purulent meningitis can be excluded. Diagnostic criteria for encephalopathy were as follows: clinical manifestations and imaging [ultrasound, computer tomography, magnetic resonance imaging (MRI)] results.

Exclusion criteria for premature infants with SAE were as follows: 1) Premature infants who did not meet the diagnostic criteria for sepsis. 2) Central nervous system infection; results of CSF examination as follows: WBC > 20 × 10^6^/L, protein > 1500 mg/L, glucose < 50% of the trace glucose in synchronous peripheral blood, and simultaneous blood culture and/or positivity. 3) No clinical manifestation of encephalopathy or brain injury supported by cranial imaging. 4) Severe perinatal asphyxia, congenital malformation, metabolic disease (neonatal hypoglycemia, congenital hypothyroidism, etc.), intracranial hemorrhage, genetic disease (e.g., birth defects such as primary immune deficiency or blood system disease).

### Gene expression microarray analysis

Peripheral blood samples (2 mL) were collected from the 20 premature infants and total RNA extracted using an RNA extraction kit (Takara Bio, Inc., Otsu, Japan). The application process of gene expression microarray analysis includes: (1) Preparation target: extract nucleotides from biological samples and label them; (2) Hybridization: incubate the target with the cDNA or oligonucleotide sequence on the chip; (3) Data acquisition: the signal intensity of the target hybridized with the probe; (4) Data analysis: draw conclusions of biological significance from a large number of data.

Analyses of RNA quantity and quality were performed using a NanoDrop ND-2000 Spectrophotometer (version 3.1). Analyses of gene expression were performed using the Agilent human 4x44K gene expression microarray v2 chip (Agilent Technology, version 2.0). According to Agilent One-Color Microarray (Agilent Technology, version 6.9.1)-Based Gene Expression Analysis Protocol, sample labelling and chip hybridization were performed using Gene Expression Analysis experimental scheme. After hybridization and washing, the microarray slides were scanned with an Agilent DNA microarray scanner. The results of mRNA expression were extracted from Agilent Feature Extraction Software (version 11.0.0.1), then imported into the Agilent.

For further analysis, we used GeneSpring GX software (version 12.1). Using Axon GenePix 4000B to scan miRNA chip and GenePix Pro 6.0 software to read probes’ signal. Metascape analysis (http://metascape.org/) was used for the gene enrichment analysis. Using DAVID Functional Annotation Bioinformatics Microarray Analysis and miRCURY NA™ microRNA Array software (Exiqon v8.1) to identify the differential expressed miRNA and its potential target genes (service provided by Kangchen Biotech, Shanghai, China). Differently expressed genes were then identified through Fold Change (FC ≥2) and *P*-value (*P*-value ≤0.05).

The following online prediction websites were used to identify miRNA target genes: miRBase, MicroCosm, TargetScan, miRDB, and miRanda. Target genes of differentially expressed miRNAs were detected by qRT-PCR.

### Evaluation of miRNA function

A literature review showed that the occurrence of brain damage in premature infants after sepsis may be related to dysfunction of mitochondrial oxidative metabolism in nerve cells. Therefore, we selected two differentially expressed miRNAs involved in regulation of the oxidative respiratory chain, based on computational prediction of differentially expressed miRNA function.

The effects of expression changes of selected miRNAs (miRNA-1197 and miRNA-485-5p) on cellular biological functions were evaluated. Lentiviruses overexpressing miRNA-1197 (LV-hsa-miR-1197; 25,200–1) and silencing miRNA-485-5p (lentivirus LV-hsa-miR-485-5p-inhibition; 25,193–1) were constructed. The effects of high miRNA-1197 expression or low miRNA-485-5p expression on the human glial cell line, U251, were determined by transfecting cells with the recombinant lentiviruses and then assessing cell proliferation using the cell counting kit-8 (CCK-8) assay and apoptosis by single staining with Annexin V-APC.

### Total RNA isolation and real-time q-PCR verification

According to the manufacturer’s instructions, we used TRIzol reagent (Invitrogen, Carlsbad, USA) to isolate total RNA from infants’ blood samples (SAE infants and non- SAE infants). RNA was quantified by NANO drop ND-2000 specttropotomete (NanoDrop Wilmington DE, version 3.1). Next, reverse transcription (RT) reactions and real-time PCR were carried out in accordance with the methods described previously. The expression coefficient of miRNA-1197, miRNA-485-5p, *ATP6V0C* and *ATP6V1C2* relative to β-actin was calculated using the 2^-ΔΔCt^ method.

### Statistical analysis

SPSS 26.0 statistical software was used to analyze the data. The data of measurement data was conformed to the normal distribution through the normal test. Experimental data were analyzed using the Student’s t-test (*p < 0.05, **p < 0.01). Throughout the paper, values are presented as the mean ± standard deviation of at least three independent experiments.

## Results

### Patient characteristics

The 20 premature infants included in this study comprised 12 males and 8 females, with mean gestational age 31.0 ± 2.46 weeks. Seven cases had birth weight ≤ 1500 g and 13 cases birth weight 1500–2500 g. Blood culture was positive in 14 cases, including 10 cases of Gram-negative bacteria (*Escherichia coli*, *Enterobacter cloacae*, *Proteus*.) and 4 cases of Gram-positive bacteria (*Streptococcus agalactiae*, *Staphylococcus aureus*, *Clostridium perfringens.*); blood culture was negative in the remaining 6 cases.

In the SAE group, comprising 10 premature infants, 7 cases were mechanically ventilated and 3 cases received an air-oxygen mixture via nasal catheter. Among the 10 non-SAE premature infants, 2 cases were mechanically ventilated, 4 cases received air-oxygen mixture via nasal catheter, and 4 cases had no history of oxygen inhalation or oxygenation (mixed air-oxygen via nasal catheter inhalation, inhaled oxygen concentration < 30%, oxygen time < 3 days), and did not develop SAE.

### Differences in mRNA and miRNA expression levels in premature infants with and without SAE

**Fig. 1:**
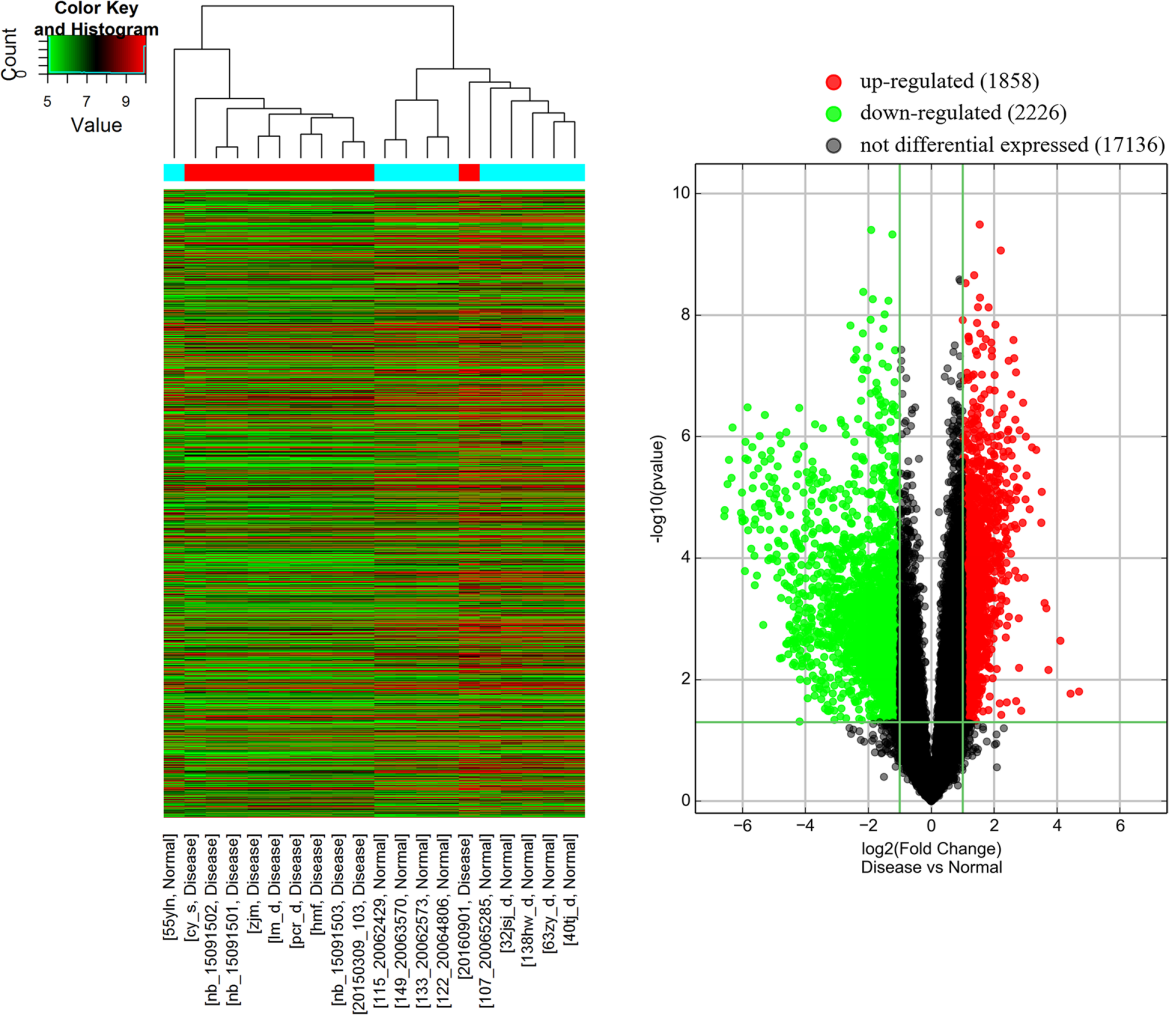
Heat map and volcanic map of differentially expressed genes between preterm infants with SAE and preterm infants without encephalopathy after sepsis. The x-axis represents the samples, and genes are shown on the y-axis. Red spots represent highly expressed genes, and green spots represent poorly expressed genes. The sample types are shown with bar colours in the dendrogram; red stripes represent samples from preterm infants with SAE, and green stripes are samples from preterm infants without encephalopathy after sepsis. Green stripes are downregulated genes in preterm infants with SAE compared with preterm infants without encephalopathy after sepsis; red stripes are upregulated genes in preterm infants with SAE

**Fig. 2:**
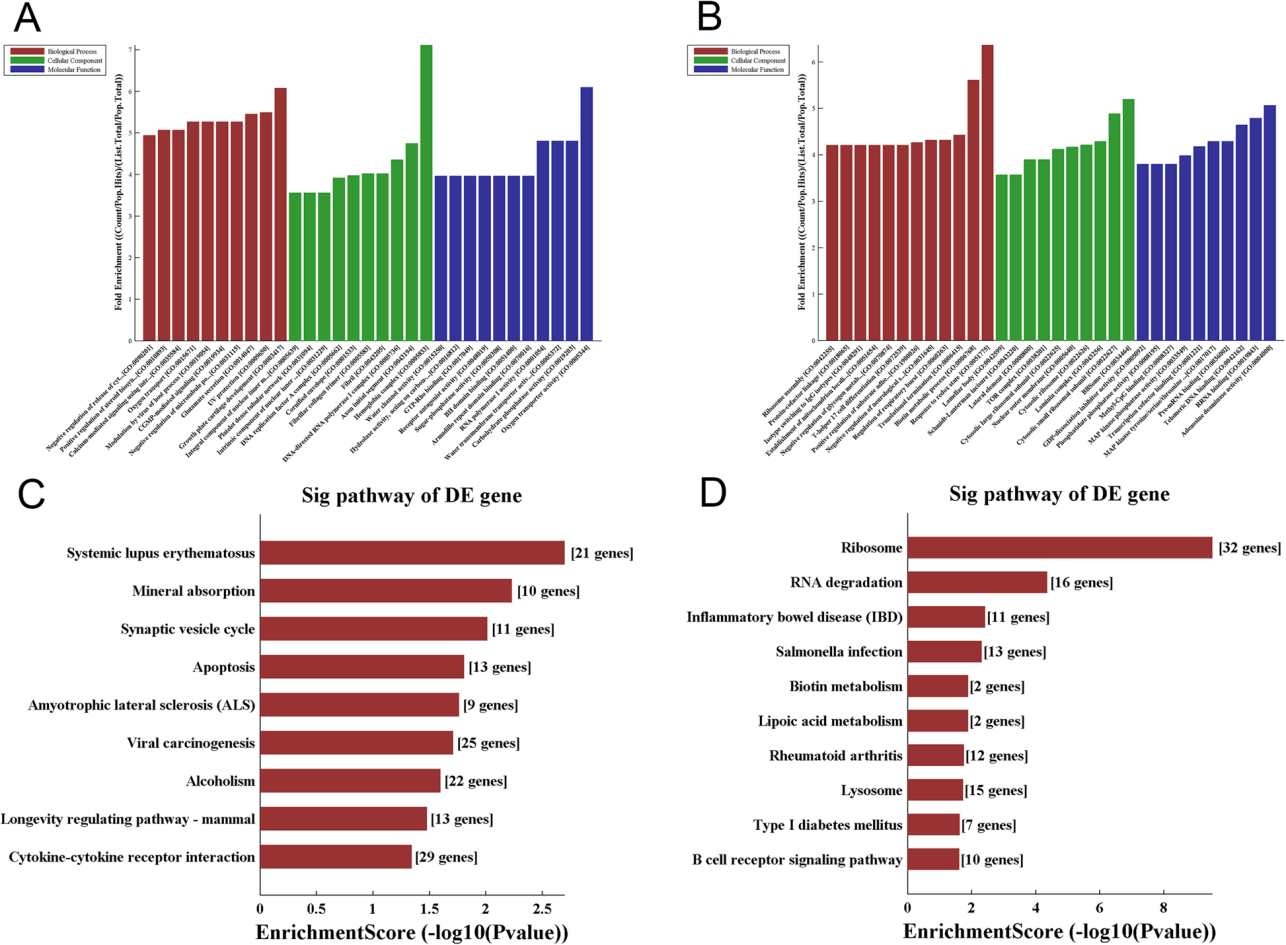
**A** Gene Ontology (GO) [Metascape analysis (http://metascape.org/)]annotation histogram of downregulated gene enrichment. **B** GO annotation histogram of upregulated gene enrichment. Red represents the type of enrichment at the BP level of GO, green represents the type of enrichment at the CC level, and blue represents the type of enrichment at the MF level. BP: Gene ontology biological processes, biological processes completed through a variety of molecular activities; CC: Gene ontology cellular component, the cell structure position of gene products when performing functions; MF: Gene ontology molecular function, the activity of a single gene product (including protein or RNA) or a complex of multiple gene products at the molecular level. **C** Kyoto Encyclopedia of Genes and Genomes (KEGG) annotation histogram of downregulated gene enrichment. **D** KEGG annotation histogram of upregulated gene enrichment. The vertical coordinate represents the KEGG pathway enriched by differentially expressed genes, and the horizontal coordinate indicates the degree of enrichment of differentially expressed genes. The higher the score, the higher the concentration

**Fig. 3:**
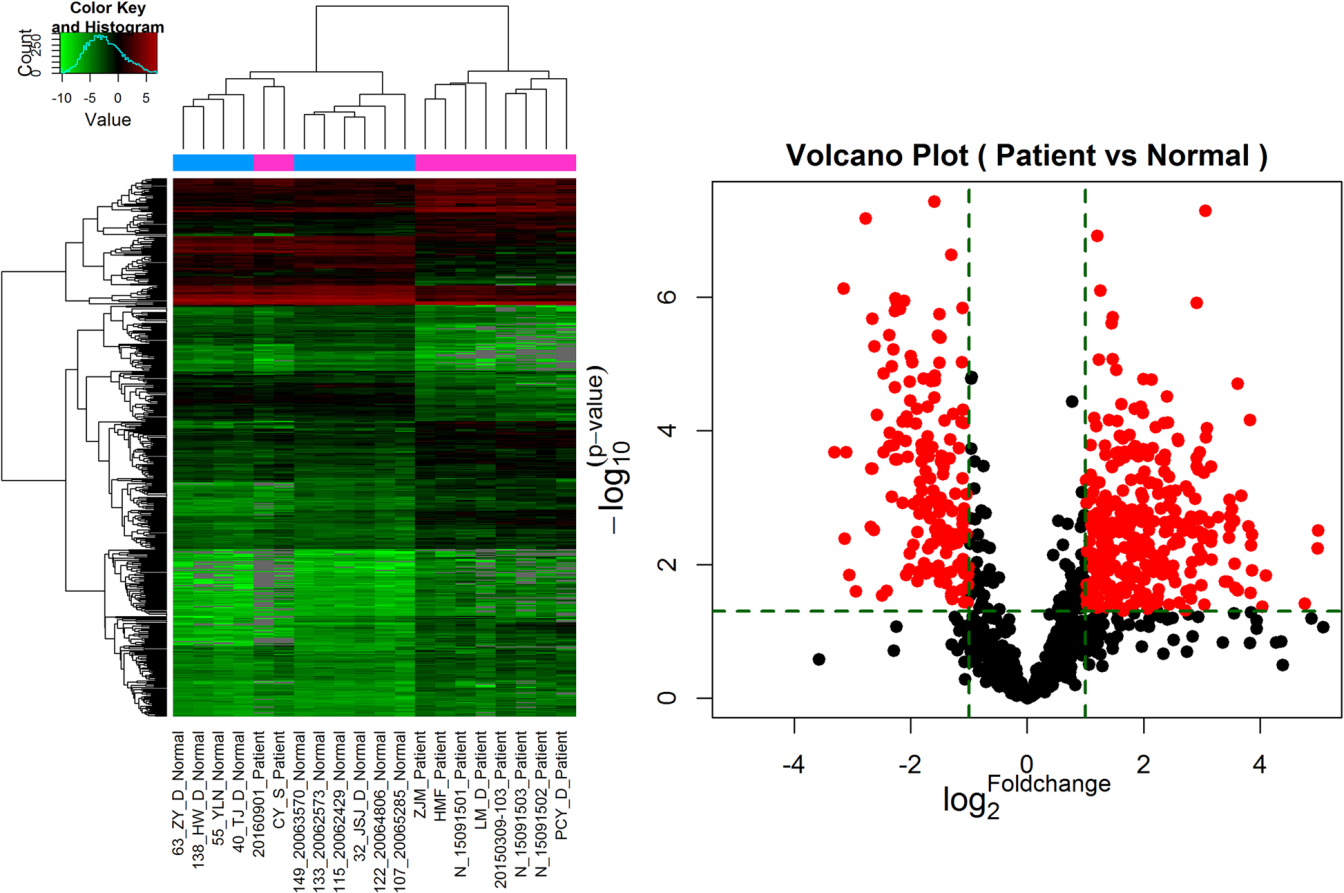
Heat map and volcanic map of differentially expressed miRNAs between preterm infants with SAE and preterm infants without encephalopathy after sepsis. In the heat map, greens pots are downregulated miRNAs and reds pots are upregulated miRNAs in preterm infants with SAE compared with preterm infants without encephalopathy after sepsis. In the volcanic diagram, the red points in the upper left corner are down regulated miRNAs and the red points in the upper right corner are upregulated miRNAs in preterm infants with SAE compared with preterm infants without encephalopathy after sepsis

Compared with preterm infants without encephalopathy after sepsis, preterm infants with SAE had 1858 significantly upregulated and 2226 significantly downregulated mRNAs with FC ≥ |2|; *p <* 0.05 (Fig. 1; Fig. 2A–D). Further, preterm infants with SAE had 322 significantly upregulated and 160 significantly downregulated miRNAs compared with the non-SAE group (FC ≥ |2|; *p* ≤ 0.05) (Fig. 3).

**Fig. 4:**
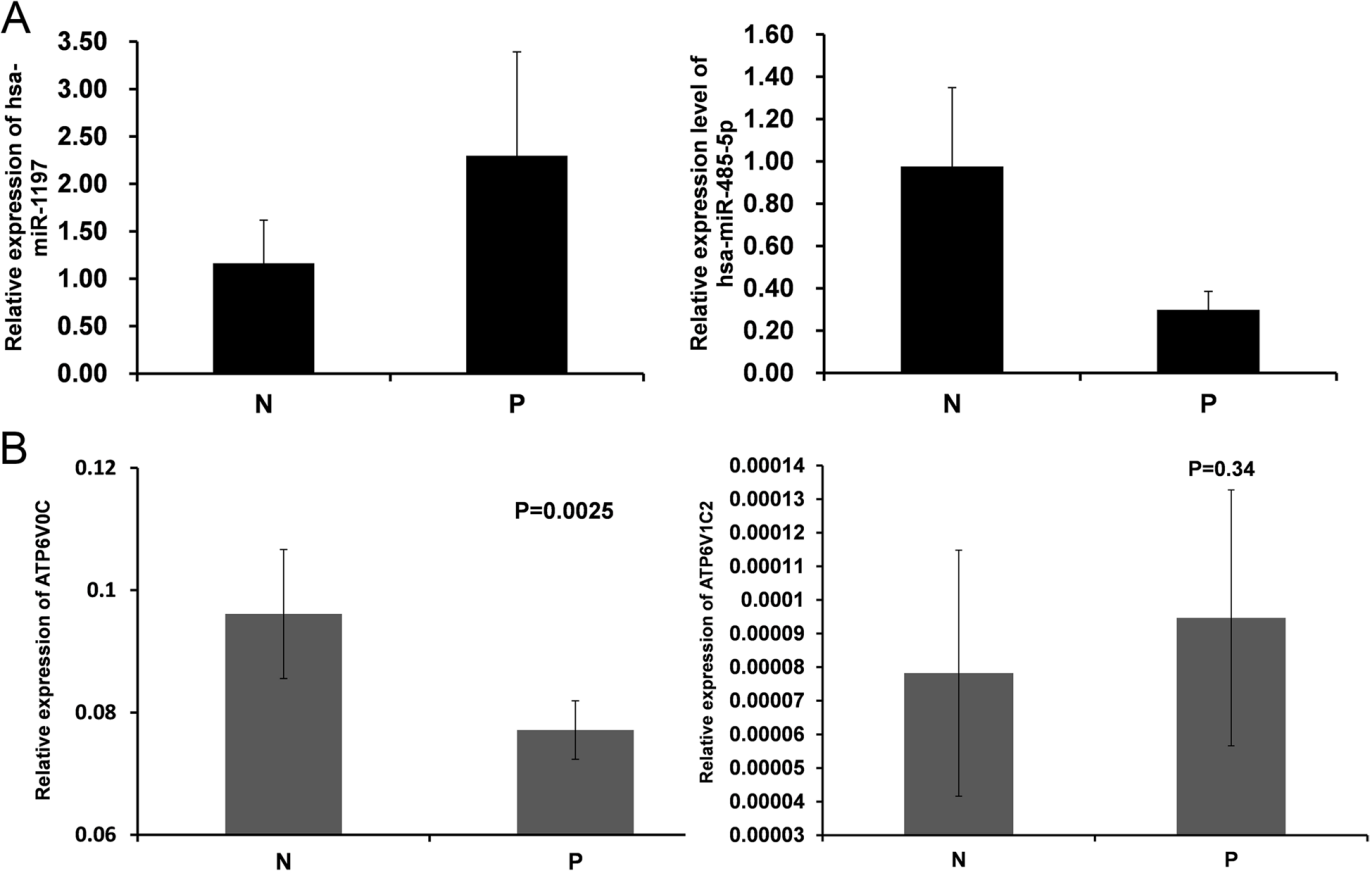
**A** qRT-PCR validation results of differentially expressed miRNA-1197 and miRNA-485-5p, which are related to the mitochondrial respiratory chain. N: preterm infants without encephalopathy after sepsis, P: preterm infants with SAE, ** *p* < 0.01. **B** qRT-PCR validation results of miRNA-1197 and miRNA- 485-5pdifferentially expressed target genes related to the mitochondrial respiratory chain. N: preterm infants without encephalopathy after sepsis, P: preterm infants with SAE, ** *p* < 0.01

Of these, miRNA-1197 (95% CI, 0.042 to 0.166) was significantly upregulated (6.03-fold) in the SAE group, while miRNA-485-5p (95% CI, 0.064 to 0.024) was significantly downregulated (0.31-fold), relative to premature infants without encephalopathy after sepsis (Fig. 4A–B).

Target genes of miRNA-1197and miRNA-485-5p identified by literature review, V0 subunit C of vacuolar ATPase (*ATP6V0C*) and *ATP6V1C2*, respectively, were selected for further analysis by qRT-PCR in infants with and without SAE. The results showed that relative expression levels of *ATP6V0C*, were significantly downregulated in the premature SAE group (*p* = 0.025), in contrast with miRNA-1197 expression levels. Further, the miRNA-485-5p target gene, *ATP6V1C2*, was downregulated in the premature SAE group, in contrast to miRNA-485-5p; however, the results were not significant (*p* = 0.34) (Fig. 4B).

### Analysis of miRNA-1197 and miRNA-485-5p function in U251 human glial cells

**Fig. 5:**
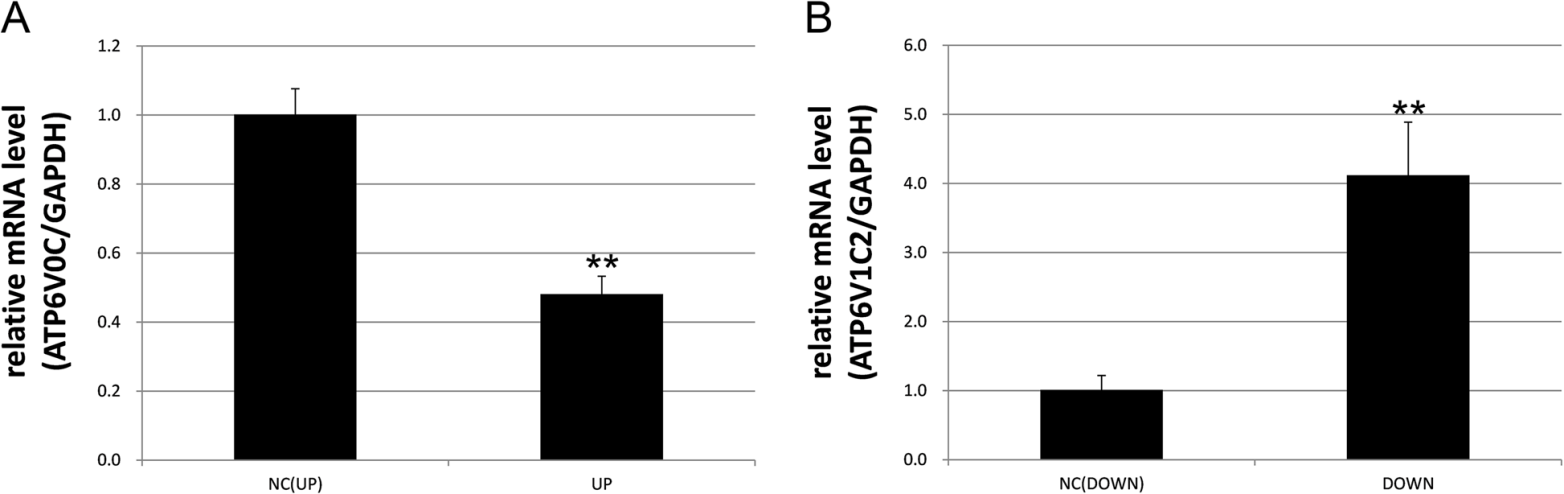
qRT-PCR validation results of target genes with high expression of miRNA-1197 and low expression of miRNA-485-5p. **A** The relative expression level of ATP6V0C, the target gene of miRNA-1197, NC is the blank lentivirus vector transfection control, and up indicatesthe lentivirus overexpressing miRNA-1197, **p < 0.01. **B** The relative expression level of ATP6V1C2, the target gene of miRNA-484-5p, NC is the blank lentivirus vector transfection control, and down indicates the lentivirus overexpressing miRNA-484-5p, **p < 0.01

Overexpression of miRNA-1197 in U251 cells led to a significant decrease in mRNA expression of *ATP6V0C*, levels of which were 0.482-fold higher in infants with SAE than in preterm infants without encephalopathy after sepsis. Further, silencing of miRNA-485-5p resulted in a significant increase in the expression of the target gene, *ATP6V1C2*, levels of which were 4.121-fold higher in infants with SAE than that in preterm infants without encephalopathy after sepsis (*p* < 0.05) (Fig. 5A, B).

## Discussion

SAE is a common, but poorly understood, neurological complication of sepsis [6] characterized by diffuse brain dysfunction, secondary to infection elsewhere in the body, without overt central nervous system infection, and often indicates a deterioration of sepsis, which has a high risk of death [7].

SAE was first described by Bright in 1827, and is an important cause of increased morbidity and mortality of patients in the ICU [8]. Further analysis showed that, even in patients with sepsis in the central nervous system and blood with no bacterial invasion, brain dysfunction can still occur [9]; this condition is caused by infection of the body, which produces a systemic inflammatory response that leads to inflammatory reactions in the brain, microcirculation, and BBB, as well as neurotransmitter transportation anomalies. Wilson et al. [10] termed this type of encephalopathy “SAE”: diffuse brain dysfunction caused by sepsis. The vast majority of diffuse brain dysfunction is reversible [11].

Although SAE is common in the ICU, the exact pathogenesis underlying the condition remains unclear and no effective treatments are available. At present, SAE is a major problem in clinical medicine, and has attracted extensive attention from researchers in China and elsewhere [12]. Although SAE pathogenesis has not been fully elucidated, current research indicates that blood–brain barrier dysfunction, brain microcirculation disorder, neuroinflammatory responses, abnormal amino acids and neurotransmitters, mitochondrial dysfunction, oxidative stress, and apoptosis likely contribute to this condition [13].

Recent years, miRNA has been considered as key biological regulators in many cell types and plays an important role in regulating gene expression, cell cycle and biological development timing [14]. In view of the small size of miRNA and easy to pass through BBB, miRNA has been proved to be excellent biomarkers for SAE diagnosis and prognosis in brain pathologies. Osca verdegal et al. [15] reported that miRNA-370 induces cell cycle arrest by targeting β- Catenin that regulates gene transcription, and miRNA-370 is the most characteristic biomarker associated with SAE.

In this study we used gene chip technology to compare mRNA and miRNA profiles in the peripheral blood of premature infants with and without SAE after sepsis and conducted a preliminary analysis of the biological functions of differentially expressed mRNAs and miRNAs, including assessment of the effects of the differentially expressed miRNAs on apoptosis in vitro. Together with a literature review, target genes of miRNA-1197 and miRNA-485-5p, *ATP6V0C* and *ATP6V1C2*, respectively, were selected for further analysis by qRT-PCR in infants with and without SAE. Expression levels of *ATP6V0C* were significantly downregulated in the premature SAE group (*p* = 0.025), in contrast with miRNA-1197 expression levels. Further, *ATP6V1C2*, was downregulated in the premature SAE group, in contrast to miRNA-485-5p; however, the results were not significant (*p* = 0.34) (Fig. 4B). ATP6V0C, is a key transmembrane component of V-ATPase, an ATP-dependent proton pump, which function is to maintain the relative neutral environment in the cell and the relative acidic environment between the cell vesicle cavity and outside the cell, by transporting H^+^ ions into or out of cells against a concentration gradient. Therefore, we speculate that energy metabolism disorder and abnormal H^+^ pump function may have important roles in the pathogenesis of SAE in premature infants.

**Fig. 6:**
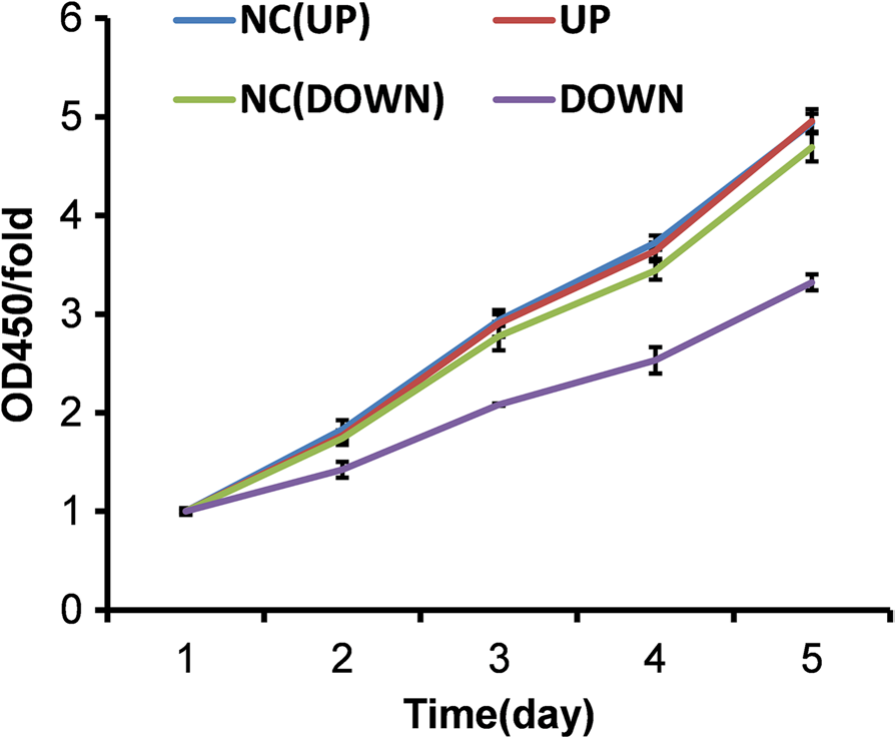
CCK-8 assay results of lentiviral transfection of U251 cells on different days; OD450/fold indicates the relative proportion of active cells, with the x-coordinate being the number of culture days

Three days after lentiviral transfection of U251 cells, the level of apoptosis of cells expressing high levels of miRNA-1197 or low levels of miRNA-485-5p, was detected by Annexin V-APC single-staining and flow cytometry (Supplemental Fig. S1). The results indicated that neither miRNA-1197 nor miRNA-485-5p expression levels significantly influenced U251 cell apoptosis, suggesting these miRNAs likely do not influence the biological function of U251 cells by affecting apoptosis (Fig. 6; Supplementary Fig. 1, 2). Our findings suggest that, when miRNA-485-5p expression is low, cell proliferation is reduced, while there is no significant effect on apoptosis. Further, miRNA-1197 had no significant effect on either cell proliferation or apoptosis, suggesting that SAE in premature infants is associated with high miRNA-1197 and low miRNA-485-5p expression. The two miRNAs selected for further study are involved in regulation of oxidative respiratory chain function and related to energy metabolism. The low expression of miRNA-485-5p observed in premature infants with SAE may lead to reduced proliferation of neuroglial cells. These two miRNAs do not appear to be involved in the occurrence of premature SAE by regulating neuroglial cell apoptosis. Chen et al. reported that the miR-485-5p/tumor necrosis factor receptor type 1-associated death domain protein (TRADD) axis participated in hydrogen sulfide-mediated protection against TNF-α-induced neuronal cell apoptosis [16].

SAE should be considered after sepsis if premature infants have consciousness disorders that do not correspond to the degree of sedation, abnormal sleep/wake cycles, hallucinations, or restlessness, and the diagnosis of SAE should be based on the following three factors [17]: (1) a systemic inflammatory reaction caused by infection (definite or highly probable) in premature infants, (2) a disturbance of consciousness that does not correspond to the degree of sedation and acute changes in the sleep/wake cycle, and (3) exclusion of primary brain diseases (such as periventricular leukomalacia, and peri/intraventricular hemorrhages) and intracranial infection in premature infants. There are no specific treatments for premature infants with SAE. Conventional treatments mainly target sepsis, to maintain the stability of the internal physiological environment and avoid brain hemodynamic disorder, and involve cephalic imaging evaluation (B ultrasound, MRI), individualized developmental support nursing, and post-discharge follow-up [18].

Premature infant SAE is a common clinical syndrome in the NICU that significantly reduces long-term cognitive function and quality of life [19, 20]. Clinical diagnosis and treatment of premature infant SAE have not improved significantly, because the pathophysiological mechanisms underlying premature infant SAE are complex, and the core mechanism involved has not been elucidated; thus, these processes require further study [21, 22].

## Conclusions

In summary, high expression of miRNA-1197 and low expression of miRNA-485-5p were strongly associated with SAE in premature infants. Both of these molecules may contribute to SAE pathogenesis in premature infants, by regulating the oxidative respiratory chain and energy metabolism. This clarifies the molecular basis of SAE in premature infants, and provides an important theoretical basis for clinical diagnosis and treatment of SAE in premature infants.

### Strengths and limitations

This study presents information regarding the molecular basis of SAE in premature infants which might assist in identifying biomarkers to circumvent such an event or better treat these infants in future. Limitations of the study include that it consisted of a small sample size from a single hospital.

## Supplementary Information

**Additional file 1: Supplemental Figure S1.** Percentage (%) of cell apoptosis in Annexin V-APC single-staining and flow cytometry. NC (up/down) is the blank lentivirus vector transfection control, up indicatesthe lentivirus overexpressing miRNA-1197, and down indicates the lentivirus overexpressing miRNA-484-5p.

**Additional file 2: Supplemental Figure S2.** Cell apoptosis of Annexin V-APC single-staining and flow cytometry. A and C: NC (up/down) is the blank lentivirus vector transfection control, B: up indicatesthe lentivirus overexpressing miRNA-1197, D: down indicates the lentivirus overexpressing miRNA-484-5p.

## Data Availability

The datasets used and/or analysed during the current study are available from the corresponding author on reasonable request.

## Abbreviations

ATP6V0C: The V0 subunit C of vacuolar ATPase
BBB: Blood-brain barrier
BBBD: Blood-brain barrier dysfunction
BP: Biological processes
CC: Cellular component
CCK-8: Cell counting kit-8
CNS: Central nervous system
CRP: Acute C-reactive protein
CSF: Cerebrospinal fluid
CT: Computer tomography
FC: Fold change
GO: Gene ontology
IL-6: Interleukin-6
KEGG: Kyoto Encyclopedia of Genes and Genomes
MF: Molecular function
miRNAs: MicroRNAs
MODS: Multiple organ dysfunction syndrome
MRI: Magnetic resonance imaging
NICU: Neonatal intensive care unit
OS: Oxidative stress
NRDS: Neonatal respiratory distress syndrome
PCT: Procalcitonin
PIVH: Peri/intraventricular haemorrhages
PVL: Periventricular leukomalacia
SAE: Sepsis-associated encephalopathy
SIRS: Systemic inflammatory response syndrome
TRADD: Tumour necrosis factor receptor type 1-associated death domain protein
WBC: White blood cell
