# Supplementary figures and images for: The molecular basis of brain injury in preterm infants with sepsis - associated encephalopathy
Source: BMC Pediatr. 2022 Jun 10;22:336. doi: 10.1186/s12887-022-03372-5 (PMC9185920; doi:10.1186/s12887-022-03372-5)

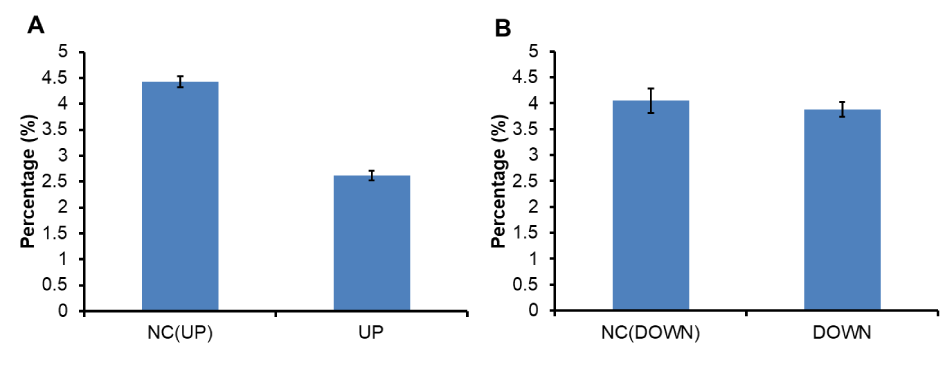

Supplement: Supplementary file 1 — Additional file 1: Supplemental Figure S1. Percentage (%) of cell apoptosis in Annexin V-APC single-staining and flow cytometry. NC (up/down) is the blank lentivirus vector transfection control, up indicatesthe lentivirus overexpressing miRNA-1197, and down indicates the lentivirus overexpressing miRNA-484-5p. [file 12887_2022_3372_MOESM1_ESM.tif]

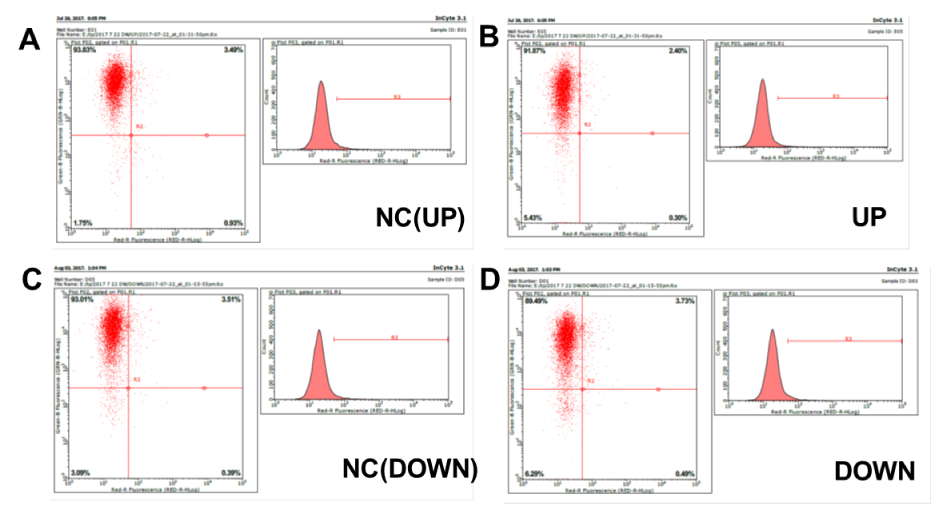

Supplement: Supplementary file 2 — Additional file 2: Supplemental Figure S2. Cell apoptosis of Annexin V-APC single-staining and flow cytometry. A and C: NC (up/down) is the blank lentivirus vector transfection control, B: up indicatesthe lentivirus overexpressing miRNA-1197, D: down indicates the lentivirus overexpressing miRNA-484-5p. [file 12887_2022_3372_MOESM2_ESM.tif]
